# Toll-Like Receptor 3 Signaling on Macrophages Is Required for Survival Following Coxsackievirus B4 Infection

**DOI:** 10.1371/journal.pone.0004127

**Published:** 2009-01-05

**Authors:** Martin J. Richer, Danielle J. Lavallée, Iryna Shanina, Marc S. Horwitz

**Affiliations:** Department of Microbiology and Immunology, University of British Columbia, Vancouver, British Columbia, Canada; Massachusetts General Hospital/Harvard University, United States of America

## Abstract

Toll-like receptor 3 (TLR3) has been proposed to play a central role in the early recognition of viruses by sensing double stranded RNA, a common intermediate of viral replication. However, several reports have demonstrated that TLR3 signaling is either dispensable or even harmful following infection with certain viruses. Here, we asked whether TLR3 plays a role in the response to coxsackievirus B4 (CB4), a prevalent human pathogen that has been associated with pancreatitis, myocarditis and diabetes. We demonstrate that TLR3 signaling on macrophages is critical to establish protective immunity to CB4. TLR3 deficient mice produced reduced pro-inflammatory mediators and are unable to control viral replication at the early stages of infection resulting in severe cardiac damage. Intriguingly, the absence of TLR3 did not affect the activation of several key innate and adaptive cellular effectors. This suggests that in the absence of TLR3 signaling on macrophages, viral replication outpaces the developing adaptive immune response. We further demonstrate that the MyD88-dependent signaling pathways are not only unable to compensate for the loss of TLR3, they are also dispensable in the response to this RNA virus. Our results demonstrate that TLR3 is not simply part of a redundant system of viral recognition, but rather TLR3 plays an essential role in recognizing the molecular signatures associated with specific viruses including CB4.

## Introduction

Protective immunity to viral infection is often critically dependent on early recognition of the pathogen. This first-line of defense against viral infection is mediated, in part, by a series of pattern-recognition receptors that include Toll-like receptors (TLRs) and Rig-like helicases (RLH). TLRs act as sensors that can recognize several molecular patterns associated with viruses including surface glycoproteins (TLR1, 2 and 4) [1]–[3] and nucleic acids (TLR3, 7, 8 and 9) [4]–[7]. Recognition of a cognate ligand induces a downstream signaling cascade culminating in the release of pro-inflammatory cytokines and maturation of antigen presenting cells (APCs). As such, TLR signaling contributes to the establishment of an anti-viral state and, by activating APCs, to the establishment of an adaptive anti-viral response.

TLR3 has long been proposed to play an important role in the innate response to viruses due to its capacity to recognize double stranded (ds) RNA, a common intermediate of viral replication [4]. Binding of dsRNA to TLR3 initiates a unique signaling cascade that, unlike other TLR ligands (with the exception of some TLR4 ligands that can initiate two separate signaling pathways) does not depend on the signaling adaptor MyD88 but rather utilizes the molecular adaptor TRIF (or TICAM-1) [8]–[10]. Despite the well documented capacity of Poly I∶C, a synthetic mimic of dsRNA, to increase antiviral immunity in a TLR3 dependent manner [11]–[16], the protective role of TLR3 *in vivo* remains controversial. For instance, a protective role for TLR3 has been demonstrated in a model of viral induced myocarditis. Following encephalomyocarditis virus (EMCV) challenge, TLR3 deficiency resulted in heightened mortality that correlated with increased viral replication and viral mediated cardiac damage. However, in this study TLR3 signaling was only partially protective as EMCV infection of wild-type (WT) mice still resulted in over 75% mortality [17]. TLR3 was also shown to play a minor role in the immune response to mouse cytomegalovirus (MCMV) [18] and murine norovirus [19] as TLR3 deficient mice were observed to harbor increased viral titers following infection although this did not affect their capacity to survive viral infection. In contrast, TLR9 deficient mice rapidly succumbed to infection by MCMV [18]. Furthermore, a dominant negative TLR3 allele was found in patients suffering from Herpes Simplex encephalitis implying a role for this receptor in protection from neurotropic viruses [20]. TLR3 signaling may be of particular importance for the immune responses to viruses that do not directly infect dendritic cells (DCs) as it was demonstrated to promote cross-priming of CD8 T cells in response to dsRNA [21]. Conversely, Edelmann and colleagues have reported that in the absence of TLR3 signaling, mice can still mount a protective immune response following challenge with various viruses, including lymphocytic choriomeningitis virus (LCMV), vesicular stomatitis virus (VSV), MCMV, and reovirus, resulting in unaffected viral clearance and no increase in viral pathogenesis [22]. Further, it was reported that TLR3 does not play a major role in the immune response of astrocytes to Theiler's murine encephalomyelitis virus (TMEV) [23]. Intriguingly, TLR3 has been observed to play a deleterious role towards the host following pathogen challenge in several models. TLR3 deficiency has been reported to confer a survival advantage following influenza A challenge due to a reduction in inflammation [24] and was also determined to protect mice following challenge with lethal doses of Punta Toro Virus by reducing viral-induced liver pathogenesis [25]. Furthermore, TLR3 deficiency was shown to be beneficial following West Nile virus infection. It was demonstrated that while the absence of TLR3 signaling resulted in increased peripheral viral load it also served to reduce neural inflammation leading to a decrease in both viral loads and pathology in the brain [26]. Conversely, a recent report has suggested that TLR3 may act to protect from West Nile virus infection directly within neurons [27]. Taken together, these data demonstrate that TLR3 may only be required for the response to a specific subset of viruses. Further, multiple reports suggest that TLR3 signaling is either dispensable or even harmful following infection with other RNA viruses.

Coxsackievirus B4 (CB4) is a small, single stranded, positive sense RNA picornavirus with tropism for the pancreas, where it has been associated with induction of pancreatitis and autoimmune diabetes (reviewed in [28]), and the heart where it can cause both acute and chronic myocarditis [29]. It is estimated that coxsackieviral infections are responsible for nearly a third of all new cases of dilated cardiomyopathy a disease responsible for the majority of heart transplantations [26], [30]. Additionally, CB4 was originally isolated from a patient suffering from type 1 diabetes [31] and has been demonstrated to accelerate onset of diabetes in non-obese diabetic (NOD) mice [32], [33]. The immune response to this virus may be strongly dependent on innate mechanism as it has been previously demonstrated that SCID mice are capable of surviving infection even at high infectious doses [34]. It was previously reported that CB4 can signal through TLR4 on pancreatic islet cells [3] and that a closely related virus, CB3, signals through TLR8 on cardiomyocytes [5]. The generation of dsRNA, the ligand for TLR3, is a common by-product of the replication cycle of single stranded RNA viruses including picornaviruses like coxsackievirus and arenaviruses like LCMV. The importance of TLRs and particularly TLR3 following CB4 infection *in vivo* remains to be elucidated.

Here, we demonstrate that the early immune response to CB4 is solely dependent on TLR3 signaling as infection of TLR3 deficient but not MyD88 deficient mice had fatal consequences. We have further demonstrated that this increased mortality is due to the inability of the host to control viral replication rapidly leading to severe cardiac damage. Interestingly, TLR3 deficiency did not affect the maturation of APCs or the activation of the adaptive immune response. Rather, TLR3 deficiency decreases the production of the pro-inflammatory mediators TNF-α and CCL5 resulting in uncontrolled viral replication that outpaces the developing adaptive immune response. Most importantly, we demonstrate that adoptive transfer of WT macrophages is sufficient to protect TLR3 deficient mice from succumbing to CB4 infection indicating that TLR3 signaling on macrophages is critical for the host response to this RNA virus.

## Results

### TLR3 plays a critical role for survival following CB4 infection

**Figure 1:**
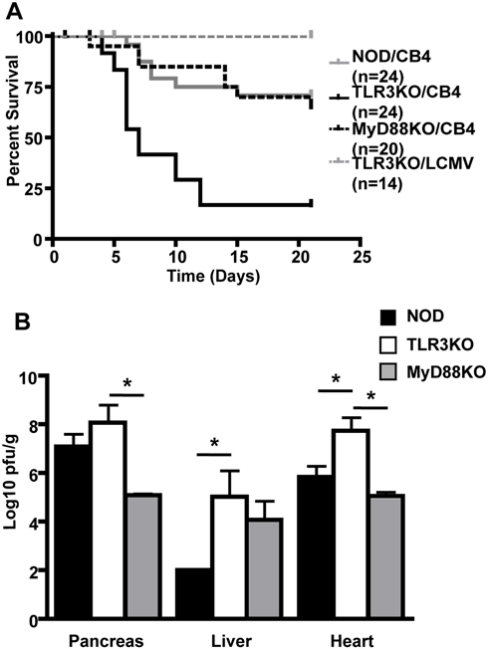
Deficiency of TLR3 results in increased mortality and increased viral replication following CB4 challenge. A) WT NOD (solid grey line), TLR3KO (solid black line) and MyD88KO mice (dashed black line) were challenged with 400 pfu of CB4 and mortality was monitored. TLR3KO mice were also challenged with 1×10^5^ pfu of LCMV (dashed grey line) as an additional control. Significantly decreased survival was observed for TLR3KO mice challenged with CB4 compared to all other mouse strains and treatment tested. B) Mice were sacrificed at day 7 PI and viral load from the pancreas liver and heart of WT NOD (black bars, n = 8), TLR3KO (white bars, n = 5) or MyD88KO (grey bars, n = 6) mice were measured by standard plaque assay. The viral titers are presented as log10 pfu/g of tissue and represent the average values of duplicates from at least 4 mice in each group. Stars denote statistical significance.

In order to elucidate the role of TLR3 in the innate immune response to coxsackievirus, NOD mice deficient for TLR3 (TLR3KO) were infected with sublethal doses of CB4 and were monitored for survival. As a control, NOD mice lacking MyD88 (MyD88KO) (and therefore unable to signal from the remaining TLRs) were also infected with CB4. We observed a significant increase in mortality following CB4 challenge in TLR3KO mice with nearly 60% of TLR3KO mice dying by day 7 compared to less than 15% of WT NOD and MyD88KO mice (Figure 1A). For comparison to other studies and to ensure that the phenotype observed was not simply dependent on mouse strain differences, mice were challenged with LCMV as TLR3 was previously demonstrated to be dispensable for the immune response to this RNA virus [22]. As previously reported, deficiency of TLR3 did not result in mortality following LCMV infection confirming that TLR3 is not necessary for the response to this virus (Figure 1A) [22]. Taken together, this suggests that while TLR3 plays a critical role in the response to some RNA viruses like CB4, it is not necessary for the control of other RNA viruses like LCMV. Importantly, the MyD88-dependent TLR signaling pathway is not required for survival following CB4 challenge, nor can it compensate for the lack of TLR3 signaling.

### Mortality is associated with increased viral replication and increased cardiac damage

**Figure 2:**
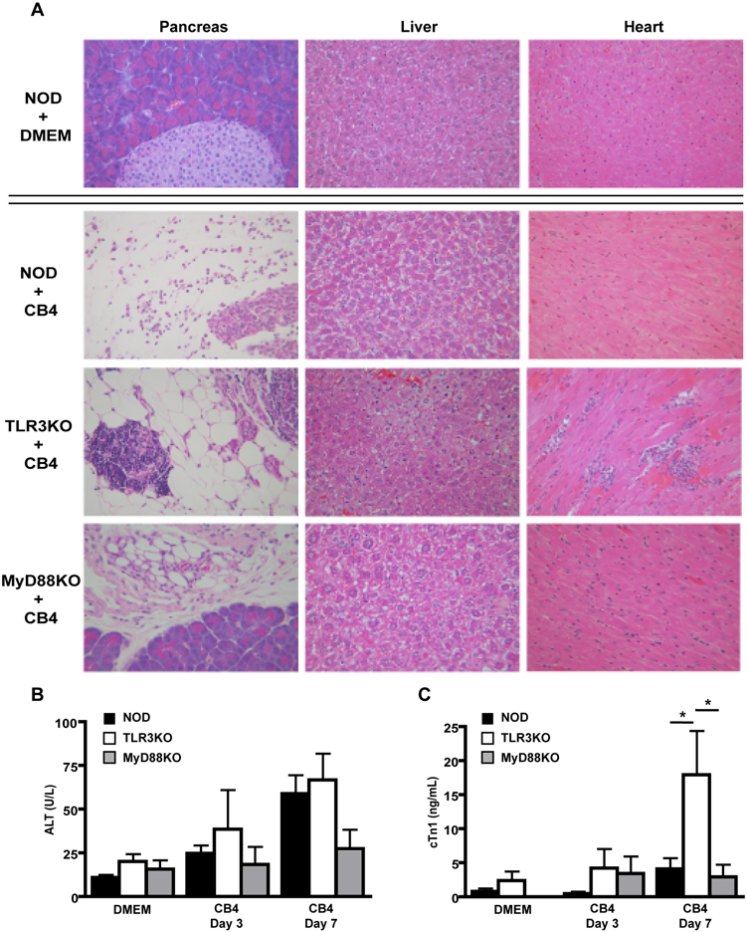
TLR3 deficiency results in increased cardiac damage during acute CB4 infection. A) Representative tissue section of pancreas (left column), liver (center column) or heart (right column) stained for H&E from mock-infected (top row) or CB4-infected WT NOD (second row), TLR3KO (third row) or MyD88KO (bottom row) mice at day 7 PI. B) Serum ALT levels of WT NOD (black bars), TLR3KO (white bars) or MyD88KO (grey bars) mice either mock-infected with DMEM or at day 3 and 7 PI with CB4. Pooled data from at least 2 independent experiments are presented as mean+/−s.e.m. C) Serum cardiac troponin 1 levels of WT NOD (black bars), TLR3KO (white bars) or MyD88KO (grey bars) mice either mock-infected with DMEM or at day 3 and 7 PI with CB4. Pooled data from at least 2 independent experiments are presented as mean+/−s.e.m . Stars denote statistical significance.

In order to determine whether increased mortality in TLR3 deficient mice resulted from an inability to control viral replication, viral titers in the pancreas, liver and heart were determined by plaque assay at day 3 and 7 post-infection (PI) with CB4. No significant differences were observed at day 3 PI (data not shown). Conversely, we observed a significant increase in viral replication in the heart and liver of TLR3KO mice compared to both WT NOD and MyD88KO mice at day 7 PI (Figure 1B). We also observed increased viral replication in the pancreas of TLR3KO mice although this difference did not reach statistical significance (Figure 1B). Most notably, at day 7 PI the viral titers in the hearts of TLR3KO mice were approximately 100 and 1000 fold higher than that observed in WT NOD and MyD88KO mice respectively (Figure 1B). We next determined whether increased viral replication resulted in heightened tissue damage that might explain the rapid death observed in TLR3KO mice. Histological examination of pancreatic tissue revealed extensive damage in TLR3KO mice that was comparable to WT NOD mice (Figure 2A). Additionally, blood glucose levels were comparable in all 3 strains at day 3 and 7 PI (data not shown) suggesting that pancreatic dysfunction is unlikely to explain the increased mortality observed. Analysis of TLR3KO livers did not reveal any obvious differences in pathology compared to either WT NOD or MyD88KO mice following CB4 infection (Figure 2A). Liver damage was further analyzed at day 3 and 7 PI using a quantitative assay based on the levels of Alanine transaminase (ALT) in the serum (Figure 2B). We observed increased ALT levels in the serum of TLR3KO mice compared to WT NOD mice at day 3 and 7 PI although this difference never reached statistical significance suggesting that liver damage is not a major contributor to the observed increased mortality in TLR3KO mice. Finally, we measured the extent of cardiac damage. Histological analysis revealed an atypical increase in the number and severity of cardiac lesions at day 7 PI in TLR3KO mice compared to both WT NOD and MyD88KO mice (Figure 2A). These observations were confirmed by a significant increase in the presence of cardiac Troponin I (cTn1), a marker for myocardial injury [35], in the serum of TLR3KO mice compared to both WT NOD and MyD88KO mice at day 7 PI (Figure 2C). Taken together, these results demonstrate that the absence of signaling from TLR3 but not other TLRs leads to uncontrolled viral replication in several organs including the heart where heightened damage is most likely responsible for the observed increase in mortality.

### TLR3 deficiency does not affect activation of innate or adaptive effectors following CB4 infection

**Figure 3:**
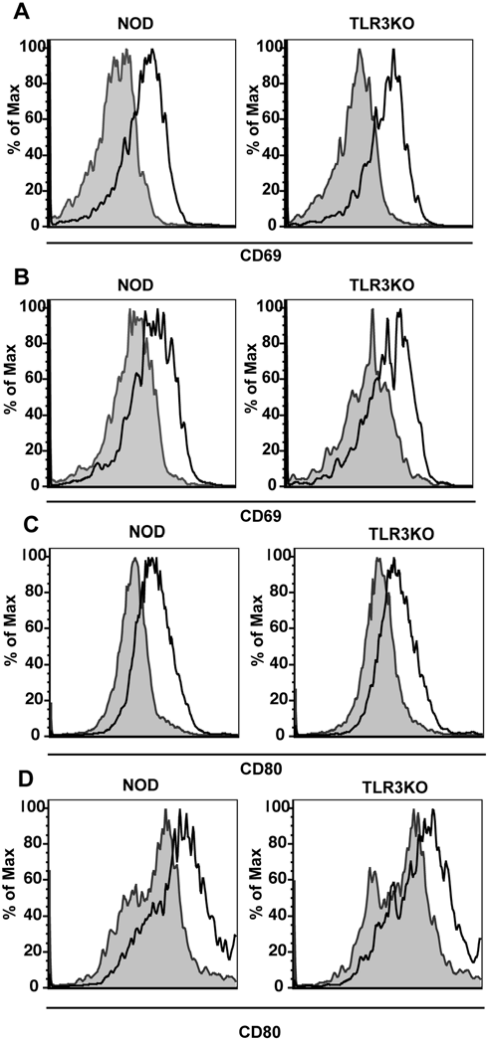
TLR3 deficiency does not affect the capacity of innate effectors to activate following CB4 infection. Representative histograms of CD69 expression on (A) NK (Pan-NK^+^, TCRβ ^−^) or (B) NK T cells (Pan-NK^+^, TCRβ ^+^) from WT NOD (left panels) and TLR3KO (right panels) mice at 48 hours post-infection with 400 pfu of CB4 (solid black lines) or mock-infection with DMEM (shaded histogram). Representative histograms of CD80 expression on the surface of (C) macrophages (CD11b+CD11c−) and (D) dendritic cells (CD11c+) from WT NOD (left panels) and TLR3KO (right panels) mice at 4 days post-infection with 400 pfu of CB4 (solid black lines) or mock-infection with DMEM (shaded histogram). Data is representative of at least 2 separate experiments.

TLR mediated signals are important for the activation of several innate effectors including NK cells and APCs. TLR signaling can also affect the activation of the adaptive immune response either directly or through effects on the APC population. As such, we sought to determine the activation status of several important antiviral effectors following CB4 infection in TLR3KO mice. First, we determined the activation of NK (pan-NK^+^ TCRβ^−^) and NK T (pan-NK^+^ and TCRβ^+^) cells by measuring surface expression of the activation marker CD69 by flow cytometry. At 48 hours post-infection, we observed that both NK and NKT cells from TLR3KO mice upregulated CD69 to levels comparable with WT NOD mice suggesting that these cells can respond normally to viral infection despite the absence of TLR3 signaling (Figure 3A, B). Interestingly, NK and NK T cell activation was completely abrogated in MyD88KO mice (Figure S1A,B). This highlights an important role for this pathway and other TLRs in the activation of NK and NK T cells although it also suggests that activation of these cells is not necessary for survival following CB4 challenge. We next determined the activation status of APCs by measuring surface expression of the costimulatory molecules CD80 (Figure 3) and CD86 (data not shown) on macrophages (CD11b+CD11c−) and DCs (CD11c+) at day 4 PI. We observed comparable upregulation of costimulatory molecules on macrophages and DCs in both WT NOD and TLR3KO mice demonstrating that TLR3 signaling is not necessary for the maturation of APCs and further suggest that a defect in APC maturation is not responsible for the increased mortality observed in TLR3KO mice (Figure 3C,D). Following infection of MyD88KO mice, we observed reduced maturation of both macrophages and DCs compared to WT mice again suggesting a role for the MyD88 pathway in the maturation of APCs (Figure S1C,D). These results suggest that the maturation of APCs is not necessary for survival following CB4 infection. As a control WT NOD and TLR3KO mice were also infected with LCMV and as expected, no differences in APC maturation were observed (Figure S2).

**Figure 4:**
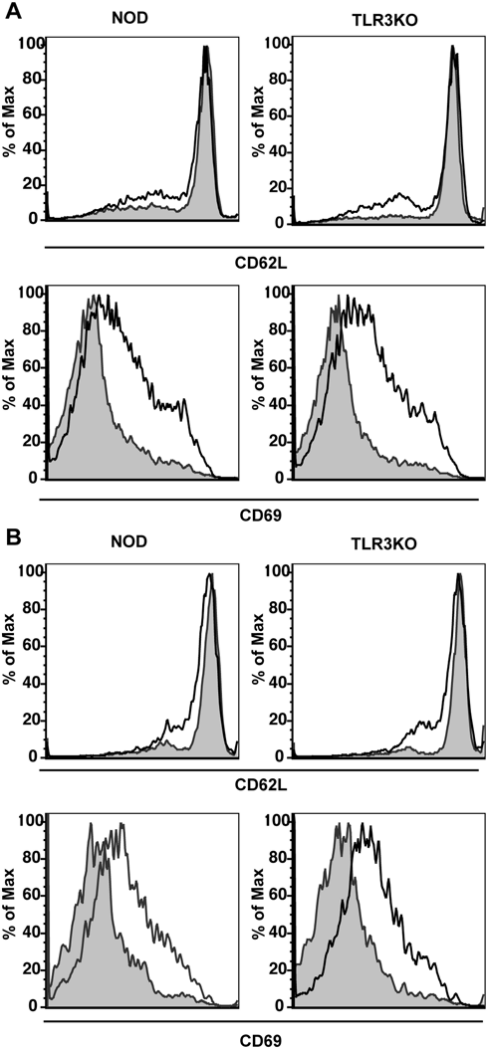
TLR3 deficiency does not affect the capacity of T cells to activate following CB4 infection. Representative histograms of CD62L and CD69 expression on the surface of (A) CD4 and (B) CD8 T cells from WT NOD (left panels) and TLR3KO (right panels) mice at 4 days post-infection with 400 pfu of CB4 (solid black lines) or mock-infection with DMEM (shaded histogram). Data is representative of at least 2 separate experiments.

To determine whether TLR3 deficiency results in changes in the activation of the adaptive immune system, the activation status of B cells and T cells was monitored by flow cytometry. At day 4 PI we observed that B cells (CD19+) from TLR3KO mice responded to viral infection by upregulating surface expression of the activation marker CD69 and the costimulatory molecule CD86 to levels comparable to that observed on B cells from WT NOD mice (Figure S3). As for innate effectors, we observed reduced activation of B cells in MyD88KO mice following CB4 challenge although as opposed to what we observed for NK cells, activation was not completely abrogated (Figure S1E). Similarly, the activation status of T cells from TLR3KO mice was comparable to WT NOD mice at day 4 PI with either CB4 (Figure 4) or LCMV (Figure S4) as measured by upregulation of CD69 and downregulation of CD62L and at day 7 (Figure S5) as measured by upregulation of CD44. Consistent with the limited maturation of APCs observed in MyD88KO mice following CB4 infection, T cell activation was also greatly reduced (Figure S1F, G). This data suggests that the maturation and activation of both innate and adaptive effectors following CB4 infection is dependent on the MyD88 signaling pathway and independent of TLR3. This further suggests that activation of cellular effectors is not critical for survival following CB4 infection.

### TLR3 deficiency results in lower production of inflammatory mediators following infection

**Figure 5:**
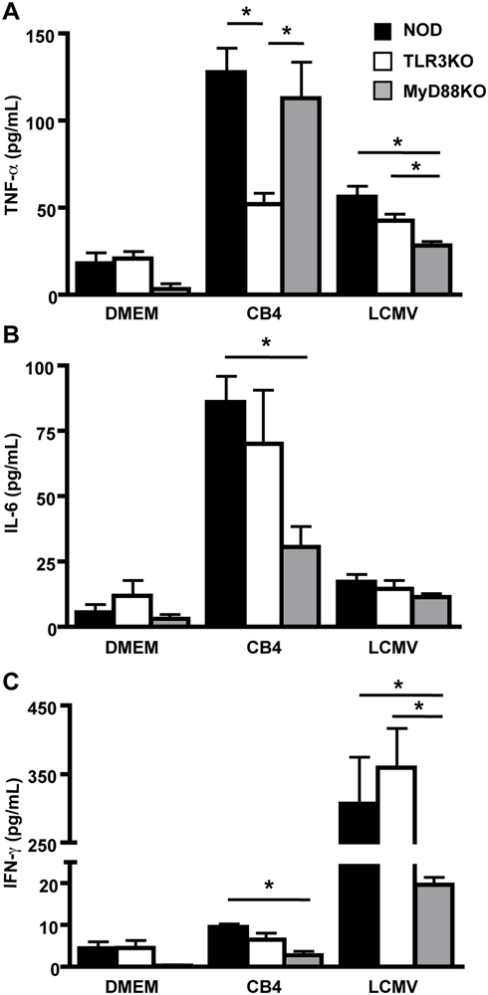
TLR3 deficiency results in reduced production of TNF-α following CB4 infection. Serum levels of A) TNF-α, B) IL-6 and C) IFN-γ from WT NOD (black bars), TLR3KO (white bars) and MyD88KO (grey bars) mice were measured with a CBA inflammation kit 4 days following infection with 400 pfu of CB4, 1×10^5^ pfu of LCMV or mock-infection with DMEM. N =  at least 3 for DMEM and LCMV treatments and n =  at least 5 mice for CB4 treatments. Pooled data from at least 2 independent experiments are presented as mean+/−s.e.m. Stars denote statistical significance.

One important functional consequence of TLR ligation with a cognate ligand is the production of pro-inflammatory cytokines and chemokines. Accordingly, we determined the serum levels of cytokines following infection. At 48 hours following infection we measured the levels of IFNα in the serum of CB4 infected mice (Figure S6), this production was abrogated in both TLR3KO and MyD88KO mice suggesting that a deficiency in type 1 interferon is unlikely to explain the increased mortality and viral replication observed in TLR3KO mice. At day 4 following CB4 infection of WT NOD mice, we observed significant increases compared to uninfected controls in the levels of both TNF-α (Figure 5A) and IL-6 (Figure 5B) and to a lesser extent IFN-γ (Figure 5C) Little to no production of IL-4, IL-5, IL-10, IL-12p70 was observed following infection (data not shown). TLR3KO mice produced significantly reduced levels of TNF-α compared to both WT NOD and MyD88KO mice while the levels of all other cytokines measured were similar to WT NOD mice (Figure 5). This suggests that production of TNF-α following CB4 infection is strongly dependent on TLR3 and independent of MyD88. Further, this suggests that TNF-α production is critical to the early immune response following infection. Interestingly, we observed a significantly reduced level of IL-6 and IFN-γ following infection of MyD88KO mice compared to WT NOD indicating that these cytokines are produced following ligation of TLRs other than TLR3 (Figure 5B,C). Further, these data suggest that IL-6 and IFN-γ do not play a critical role in the survival of mice following CB4 challenge as MyD88KO mice survived infection and controlled viral replication despite reduced levels of both of these cytokines. Following LCMV infection and consistent with prior data [36], we observed a strong cytokine response dominated by the production of IFN-γ (Figure 5C). It was previously reported that cytokine production following LCMV infection is strongly dependent on the MyD88 pathway [37], [38]. Similarly, we observed a significant reduction of IFN-γ and TNF-α production following infection of MyD88KO compared to WT NOD mice (Figure 5A,B). TLR3KO mice produced comparable levels of IFN-γ following LCMV challenge (Figure 5C) confirming that this receptor does not play a central role in the response to LCMV.

**Figure 6:**
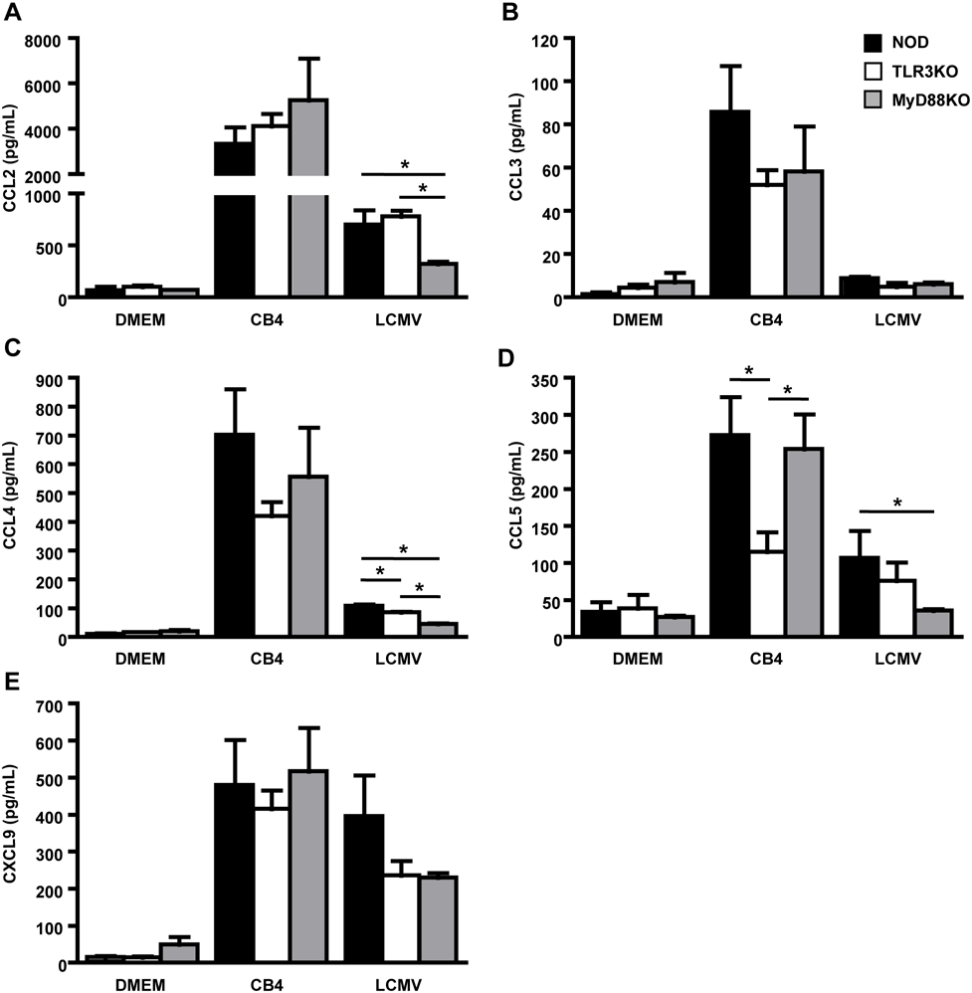
TLR3 deficiency results in reduced production of CCL5 following CB4 infection. Serum levels of A) CCL2, B) CCL3, C) CCL4, D) CCL5 and E) CXCL9 from WT NOD (black bars), TLR3KO (white bars) and MyD88KO (grey bars) mice were measured with a CBA chemokine flex set 4 days following infection with 400 pfu of CB4, 1×10^5^ pfu of LCMV or mock-infection with DMEM. n =  at least 3 for DMEM and LCMV treatments and n =  at least 5 mice for CB4 treatments. Pooled data from at least 2 independent experiments are presented as mean+/−s.e.m. Stars denote statistical significance.

Serum levels of chemokines were investigated at day 4 PI with CB4 and LCMV. Following infection significant increases of CCL2 (MCP-1), CCL3 (MIP-1α), CCL4 (MIP1-β), CCL5 (RANTES) and CXCL9 (MIG) were observed in WT NOD mice compared to uninfected controls (Figure 6). Following CB4 challenge, TLR3KO mice produced similar levels of CCL2 (Figure 6A) and CXCL9 (Figure 6E) while levels of CCL3 (Figure 6B) and CCL4 (Figure 6C) were slightly reduced but were not significantly different from either WT NOD controls or MyD88KO mice suggesting that these chemokines are not critical for survival following infection (Figure 6). Importantly, levels of CCL5 were significantly reduced in TLR3KO mice compared to both WT NOD and MyD88KO mice (Figure 6D) suggesting that production of this chemokine following CB4 challenge is strongly dependent on TLR3 and that production of this chemokine is central to the capacity of the host to control CB4 viral replication. No significant differences in chemokine production were observed between WT NOD and MyD88KO mice following infection further suggesting that the early immune response to CB4 is MyD88 independent (Figure 6). As we observed in the case of the cytokine response, the production of chemokines following LCMV infection was primarily dependent on the MyD88 pathways with MyD88KO mice producing significantly reduced levels of CCL2 and CCL5 compared to WT NOD mice. Taken together, these data demonstrate that the production of some inflammatory mediators following CB4 infection is independent of the MyD88 signaling pathway and strongly dependent on TLR3 signaling. Further, the production of TNF-α and CCL5 appear to be critical for the control of viral replication and the survival of the host following CB4 challenge.

### TLR3 signaling on macrophages plays an important role in the immune response to CB4

**Figure 7:**
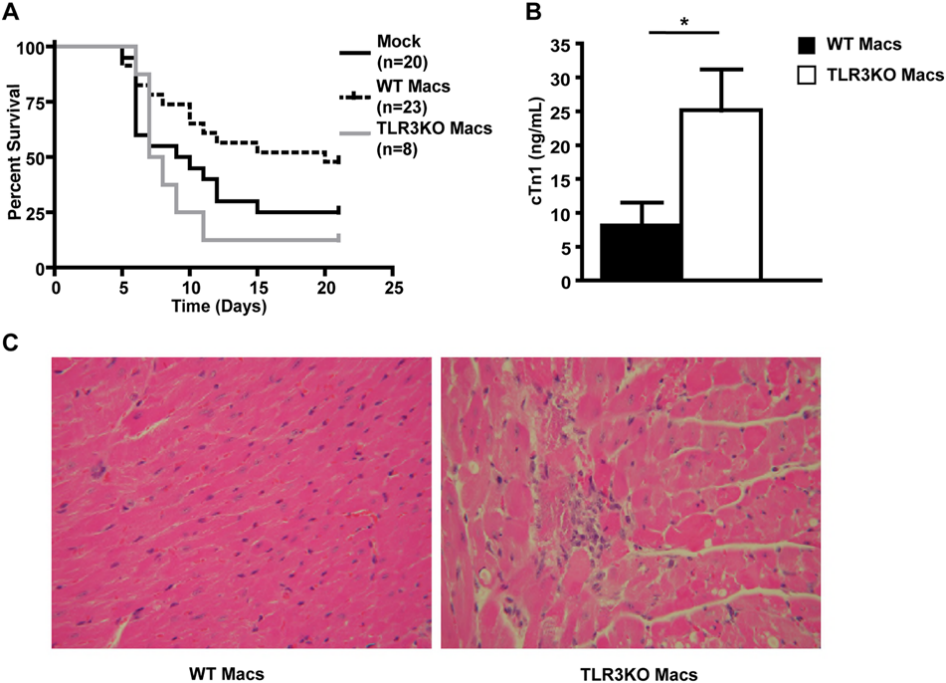
Adoptive transfer of WT macrophages rescues TLR3KO mice following CB4 challenge. A) TLR3KO mice were adoptively transferred with macrophages from WT NOD mice (dashed black line) or macrophages from TLR3KO mice (solid grey line) or mock-treated with DMEM (solid black line) and infected with 400 pfu of CB4 24 hours post-transfer. B) Serum cardiac troponin 1 levels of TLR3KO mice adoptively transferred with either WT NOD macrophages (black bars, n = 5) or TLR3KO macrophages (white bars, n = 3) at day 7 PI with CB4. Pooled data from at least 2 independent experiments are presented as mean+/−s.e.m. Stars denote statistical significance. C) Representative tissue section of day 7 PI hearts stained for H&E from CB4-infected TL3KO mice adoptively transferred with WT NOD macrophages (left panel) or TLR3KO macrophages (right panel).

Several cell types including macrophages and DCs are responsible for production of cytokines and chemokines following TLR ligation. In order to determine whether presence of TLR3 on macrophages or DCs was sufficient to decrease the susceptibility of a TLR3 deficient host, adoptive transfer experiments were performed. Splenocytes from WT NOD or TLR3KO mice were purified by flow cytometry in order to obtain two separate populations based on CD11c and CD11b expression. TLR3KO mice were adoptively transferred with a population containing either predominantly macrophages (CD11b+CD11c−) or a population containing DCs (CD11c+) and challenged with CB4 at 24 hours post-transfer. We observed that while adoptive transfer of WT DCs did not confer a survival advantage (data not shown), transfer of WT macrophages resulted in greater survival with the median survival time extended to 20 days compared to 9 days for mock treated and nearly 25% more mice surviving to 21 days PI (Figure 7A). Further, adoptive transfer of WT macrophages conferred a significant survival advantage compared to mice adoptively transferred with macrophages purified from TLR3KO donors with the median survival time extended to 20 days compared to 7.5 days and greater than 35% more mice surviving to day 21 (Figure 7A) confirming that simply increasing the number of macrophages available to respond to virus is not sufficient to confer protection. Instead, TLR3 signaling on macrophages is required to establish protection and ensure survival following CB4 infection. In addition, this suggests that TLR3 signaling on DCs is not sufficient to confer survival following CB4 infection. Importantly, after transfer of WT macrophages, we observed a significant reduction in serum levels of cTn1 (Figure 7B) as well as the number and severity of cardiac lesions in infected mice (Figure 7C) compared to infected mice adoptively transferred with macrophages from TLR3KO mice. This demonstrates that TLR3 signaling on macrophages is critical to prevent viral-mediated cardiac pathology. Furthermore, we observed that protection following adoptive transfer of WT macrophages did not correlate with increased serum levels of TNFα or CCL5 (data not shown). These results suggest that macrophages contribute to protection independent of significant increases of these mediators in the periphery and, as expected, macrophages contribute to the antiviral response in a number of ways both globally and locally within the infected tissue.

## Discussion

In this study, we demonstrate that TLR3 signaling on macrophages is required for survival following CB4 infection. Mice lacking TLR3 succumb rapidly to viral infection due to uncontrolled viral replication leading to a rapid increase in cardiac damage. Conversely, we observed that MyD88 deficiency did not result in increased mortality suggesting that signaling from other TLRs is not necessary during the early stages of CB4 infection. Our data further highlights the important role of pro-inflammatory mediators, specifically TNF-α and CCL5, in controlling viral replication in the host.

In light of the redundancy of innate receptors capable of recognizing virus-associated patterns, it is intriguing that TLR3 plays such a critical in the early response to CB4 infection. Despite the fact that coxsackieviruses have previously been described to signal through other TLRs [3], [5], our results suggests that the other TLRs as well as the other innate viral sensors such as RLHs are unable to compensate for the loss of TLR3. Additionally, our results suggest that not only are other innate viral sensors unable to compensate for the loss of TLR3, they are in fact dispensable for the early response to CB4. This is supported by a report describing that deficiency of MyD88 provided a survival advantage compared to WT mice following challenge with high infectious doses of coxsackievirus B3 [39]. These intriguing observations can be explained by several non-exclusive mechanisms. One likely explanation is that CB4 is recognized by TLRs other than TLR3 but the functional consequences of this interaction are not critical for survival. To this effect, we observed that IL-6 production was significantly reduced following CB4 infection in MyD88KO but not TLR3KO mice, suggesting that CB4 must signal through at least one MyD88-dependent pathway and that the resulting production of IL-6 is not critical for the early control of viral replication. Subcellular localization may explain why the RLHs, in particular MDA-5 which has previously been demonstrated to play an important role in the recognition of other viruses including murine norovirus and the picornavirus, EMCV [19], [40], are unable to compensate for the lack of TLR3. As opposed to TLR3 which is usually localized within endosomal compartments [21], MDA-5 is localized in the cytoplasm [41]. CB4 enters the cell by receptor-mediated endocytosis and therefore would be localized within the endosome rapidly after infection. However, as CB4 is a single stranded virus it seems unlikely that dsRNA intermediates are generated prior to the start of viral replication in the cytoplasm. Alternatively, other picornaviruses, including poliovirus and hepatitis A virus, have been demonstrated to prevent MDA-5 signaling by cleaving either MDA-5 itself or the signaling adaptor molecule mitochondrial antiviral signaling adaptor (MAVS) [42], [43]. Based on these reports, we are currently investigating the interaction between RLHs and CB4 and speculate that this virus may be able to abrogate signaling from RLHs potentially explaining the critical role of TLR3 over the course of infection.

Here, we report that TLR3 deficiency does not affect the activation of several cellular effectors typically involved in the response to viruses including NK cells and APCs. Instead, the absence of TLR3 signaling resulted in significantly reduced levels of the cytokine TNF-α and the chemokine CCL5. This suggests that early production of these inflammatory mediators plays a critical role in controlling viral replication and that, in their absence, even the activation of innate effectors and the adaptive response are insufficient to reestablish control of the viral infection. To this effect, we observed that although MyD88 mice present with reduced immune cell activation following CB4 infection, they produce similar levels of TNF-α and CCL5 compared to WT mice. The survival of these mice following CB4 infection further highlights the importance of these pro-inflammatory mediators in the control viral replication at the earliest stages of infection. Similarly, it was previously reported that SCID mice, that lack T and B cells, are able to survive the early stages of infection with CB4 suggesting that these cell types are not critical for survival [34].

TNF-α is a pro-inflammatory cytokine produced by several cell types, including macrophages, that has been ascribed antiviral properties both *in vitro* and *in vivo* (reviewed in [44]). Wada and colleagues have reported that mice lacking TNF-α succumb rapidly to EMCV infection due to increased viral replication in the heart [45], an observation that was supported following EMCV infection of TLR3 deficient mice where reduced TNF-α production was observed and correlated with increased viral replication, cardiac damage and increased mortality [17]. The antiviral effects of TNF-α may be, in part, mediated by its capacity to induce the production of nitric oxide from macrophages as this molecule has previously been demonstrated to play an important role in the control of coxsackieviral replication within the heart [46]. Taken together with the data presented in this report, this suggests that the TLR3-dependent production TNF-α plays a particularly important role in cardiac protection following infection with cardiotropic viruses.

CCL5 is a pro-inflammatory chemokine with chemotactic properties towards both T cells and monocytes/macrophages that is commonly associated with anti-viral responses. Blocking CCL5 activity has previously been demonstrated to result in increased viral antigen in the central nervous system (CNS) of mice following TMEV challenge [47]. Interestingly, normal T cell infiltration was also observed in these mice suggesting that T cells were still able to migrate to the CNS and that the antiviral activity of CCL5 is not simply due to recruitment of T cells [47]. Based on the early mortality observed in our model, it seems unlikely that improper recruitment of T cells is responsible for this increased susceptibility to CB4 infection. Rather, we hypothesize that CCL5 deficiency results in a decrease recruitment of macrophages that may be important for the production of TNF-α and, potentially, nitric oxide. To this effect, we observed that the adoptive transfer of a population composed mostly of macrophages but not DCs from WT mice was sufficient to reduce mortality induced by CB4 in TLR3KO mice by preventing viral-mediated cardiac pathology. These data suggest that TLR3 signaling on macrophages results in the production of TNF-α and CCL5 both of which act to control early viral replication which, in turn, likely allows for the adaptive immune response to activate and clear the viral infection.

Contrary to what has been reported for several other viruses including another picornavirus [22], [23], TLR3 signaling plays a role in the protection of the host from cardiotropic viruses such as coxsackievirus and EMCV[17]. Susceptibility to viral-induced myocarditis is dependent on several genetic traits [48]. Our observations suggest that genetic differences leading to changes in TLR3 function or expression could be linked to susceptibility to viral-induced myocarditis. To this effect, it was recently reported that differences in susceptibility to chronic viral-induced myocarditis between a C57Bl/6 and A.BY/SnJ mice was linked with reduced production of TNF-α and CCL5 following coxsackievirus B3 infection in a potentially TLR3-dependent manner [49]. Similarly, patients with a TLR3 deficiency are highly susceptible to Herpes Simplex encephalitis [20]. Taken together, these observations suggest that therapies aimed at re-establishing TLR3 signaling or its functional consequences may provide an effective means to protect susceptible patients from suffering the fatal consequences of viral myocarditis.

In summary, we provide evidence for an essential non-redundant role for TLR3 signaling on macrophages in the early stages of coxsackievirus B4 infection. Our results demonstrate that the production of important pro-inflammatory mediators such as TNF-α and CCL5 are dependent on the TLR3 signaling pathway and independent of the MyD88 pathway. Further, we demonstrate that mortality occurs despite the normal activation of the adaptive immune response. This suggests that in the absence of macrophages recognizing the invading pathogen in a TLR3 dependent manner, viral replication cannot be controlled and is allowed to outpace the developing adaptive immune response. The differences in requirement for TLR3 signaling between RNA viruses (eg. LCMV and CB4) suggest that the wide variety of viral-specific pattern recognition receptors does not represent simple redundancy. Rather this system has been evolved to provide a first-line of defense tailored to the requirements of the host to protect itself against a specific virus. As such, gaining understanding of this complex interplay of viruses with the innate immune system will likely provide insight for the design of more effective antiviral therapeutics.

## Materials and Methods

### Mice

NOD/ShiLtJ and TLR3KO mice were obtained from The Jackson Laboratory (Bar Harbor, USA). TLR3KO mice were backcrossed and maintained on the NOD background. MyD88KO mice on the NOD background were a generous gift from Dr. A. Chervonsky (University of Chicago). All mice were bred and maintained in our rodent facility and monitored for blood glucose prior to infection to ensure that only pre-diabetic mice were analyzed. All performed procedures followed the guidelines of the institutional animal care committee.

### Virus

Stocks of CB4 Edwards strain 2 was originally obtained from Dr. C. Gauntt (University of Texas-San Antonio) and prepared as described previously [50], [51]. LCMV Armstrong strain 53b was originally obtained from Dr. M.B. Oldstone and propagated as described previously [52]. 6–8 week old mice were infected intraperitoneally with sublethal doses of 400 PFU of CB4 or 1×10^5^ PFU of LCMV. Viral titers were measured following standard plaque assay procedures.

### Flow cytometry

Single cell suspensions were generated from the spleen at the indicated time points, stained for the appropriate markers and analyzed by flow cytometry. Fluorescently conjugated antibodies directed against CD11b (clone M1/70), CD11c (clone HL3), CD4 (clone L3T4), CD8 (clone 53-6.7), CD19 (clone eBio 1D3), panNK (clone DX5) and CD69 (clone H1.2F3) were purchased from eBiosciences (San Diego, USA) while biotin conjugated antibodies directed against CD44 (clone IM7), CD62L (clone Mel-14), CD69 (clone H1.2F3), CD80 (clone 16-10A1) and, CD86 (clone GL1) were purchased from BD Biosciences (Missisauga, Canada).

### Immunohistochemical staining

Tissue sections were prepared as previously described [51] at day 7 PI. Staining was performed using standard procedures for hematoxylin and eosin (Wax-It, Vancouver, British Columbia). Tissue sections were graded for pathology.

### Quantitative measurement of liver damage

Liver damage was quantified at days 3 and 7 PI by measuring serum levels of alanine transaminase (ALT) using commercially available Infinity™ ALT reagents (Thermo Scientific, Waltham, MA) according to manufacturer's instructions.

### Quantitative measurement of heart damage

Heart damage was quantified by measuring release of cardiac troponin 1 into the serum of mock-infected or infected mice at days 3 and 7 PI using a commercially available ELISA kit (Life Diagnostics, West Chester, PA) according to manufacturer's instruction.

### Cytokine and chemokine analysis

Serum cytokine and chemokine levels were measured at day 4 PI using a mouse inflammation CBA kit (BD Bioscience) allowing for simultaneous detection IL-6, IL-10, MCP-1, IFN-γ, TNF-α and IL-12p70 from a single sample or a chemokine flex set (BD Bioscience) customized to allow for simultaneous detection of CXCL9 (MIG), CCL3 (MIP1α), CCL4 (MIP1β) and CCL5 (RANTES) from a single sample. Samples were prepared according to manufacturer's instructions and analyzed on a BDFacsArray equipped with FCAP software (BDBiosciences). Serum levels of IFNα were measured at 48 hours PI using a VeriKine™ Mouse interferon alpha kit (PBL interferon source, Piscataway, NJ) according to manufacturer's instructions.

### Adoptive transfer experiments

Splenic single cell suspension from uninfected WT NOD or TLR3KO mice were stained with fluorescently conjugated antibodies specific for CD11b and CD11c. Cells were sorted by FACS into two separate populations composed mainly of macrophages (CD11b^+^CD11c−) or mainly dendritic cells (CD11c^+^ with varying expression of CD11b). TLR3KO mice were adoptively transferred with 2×10^5^ and a number of DCs corresponding to the natural ratio of these cells compared to the CD11b^+^CD11c− group (between 70,000 to 150,000) intraperitoneally and challenged with CB4 24 hours post-transfer.

### Statistical Analysis

Kaplan-Meier analysis was used to compare survival curves and unpaired Student's t-test was used for all other analyses. A P value of <0.05 was considered significant.

## Supporting Information

Figure S1 MyD88 deficiency decreases the capacity of cellular effectors to activate in response to CB4 infection. Representative histograms of expression of activation or costimulatory marker (as indicated) on the surface of A) NK cells (Pan-NK+, TCRβ −), B) NK T cells (Pan-NK+, TCRβ +), C) macrophages (CD11b+CD11c−), D) dendritic cells (CD11c+), E) B cells (CD19+), F) CD4+ T cells and G) CD8+ T cells from MyD88KO mice at day 4 post-infection with 400 pfu of CB4 (solid black lines) or mock-infection with DMEM (shaded histogram). Data is representative of at least 2 separate experiments.
(0.70 MB TIF)

Figure S2 TLR3 deficiency does not affect the capacity of APCs to mature following LCMV infection. Representative histograms of CD80 and CD86 expression on the surface of (A) macrophages (CD11b+CD11c−) and (B) dendritic cells (CD11c+) from WT NOD (left panels) and TLR3KO (right panels) mice at 4 days post-infection with 1×105 pfu of LCMV (solid black lines) or mock-infection with DMEM (shaded histogram). Data is representative of at least 2 separate experiments.
(0.59 MB TIF)

Figure S3 TLR3 deficiency does not affect the capacity of B cells to activate following CB4 infection. Representative histograms of CD69 and CD86 expression on the surface of B cells (CD19+) from WT NOD (left panels) and TLR3KO (right panels) mice at 4 days post-infection with 400 pfu of CB4 (solid black lines) or mock-infection with DMEM (shaded histogram). Data is representative of at least 2 separate experiments.
(0.33 MB TIF)

Figure S4 TLR3 deficiency does not affect the capacity of T cells to activate following LCMV infection. Representative histograms of CD62L and CD69 expression on the surface of (A) CD4 and (B) CD8 T cells from WT NOD (left panels) and TLR3KO (right panels) mice at 4 days post-infection with 1×105 pfu of LCMV (solid black lines) or mock-infection with DMEM (shaded histogram). Data is representative of at least 2 separate experiments.
(0.61 MB TIF)

Figure S5 TLR3 deficiency does not affect T cell activation following LCMV or CB4 infection. Representative histograms of CD44 expression on the surface of (A, C) CD4 and (B, D) CD8 T cells from WT NOD (left panels) and TLR3KO (right panels) mice at 7 days post-infection with (A,B) 400 pfu of CB4 (solid black lines) or (C,D) 1×105 pfu of LCMV (solid black lines) or mock-infection with DMEM (shaded histogram). Data is representative of at least 2 separate experiments.
(0.61 MB TIF)

Figure S6 Type 1 interferon production is reduced following CB4 infection in both TLR3KO and MyD88KO mice. Serum levels of IFNα from WT NOD (black bars), TLR3KO (white bars) and MyD88KO (grey bars) mice were measured with a VeriKine Elisa Kit at 48 hours following infection with 400 pfu of N =  at least 7 for each group. Pooled data from at least 2 independent experiments are presented as mean+/−s.e.m.
(0.20 MB TIF)
