# Supplementary figures and images for: Toll-Like Receptor 3 Signaling on Macrophages Is Required for Survival Following Coxsackievirus B4 Infection
Source: PLoS One. 2009 Jan 5;4(1):e4127. doi: 10.1371/journal.pone.0004127 (PMC2606033; doi:10.1371/journal.pone.0004127)

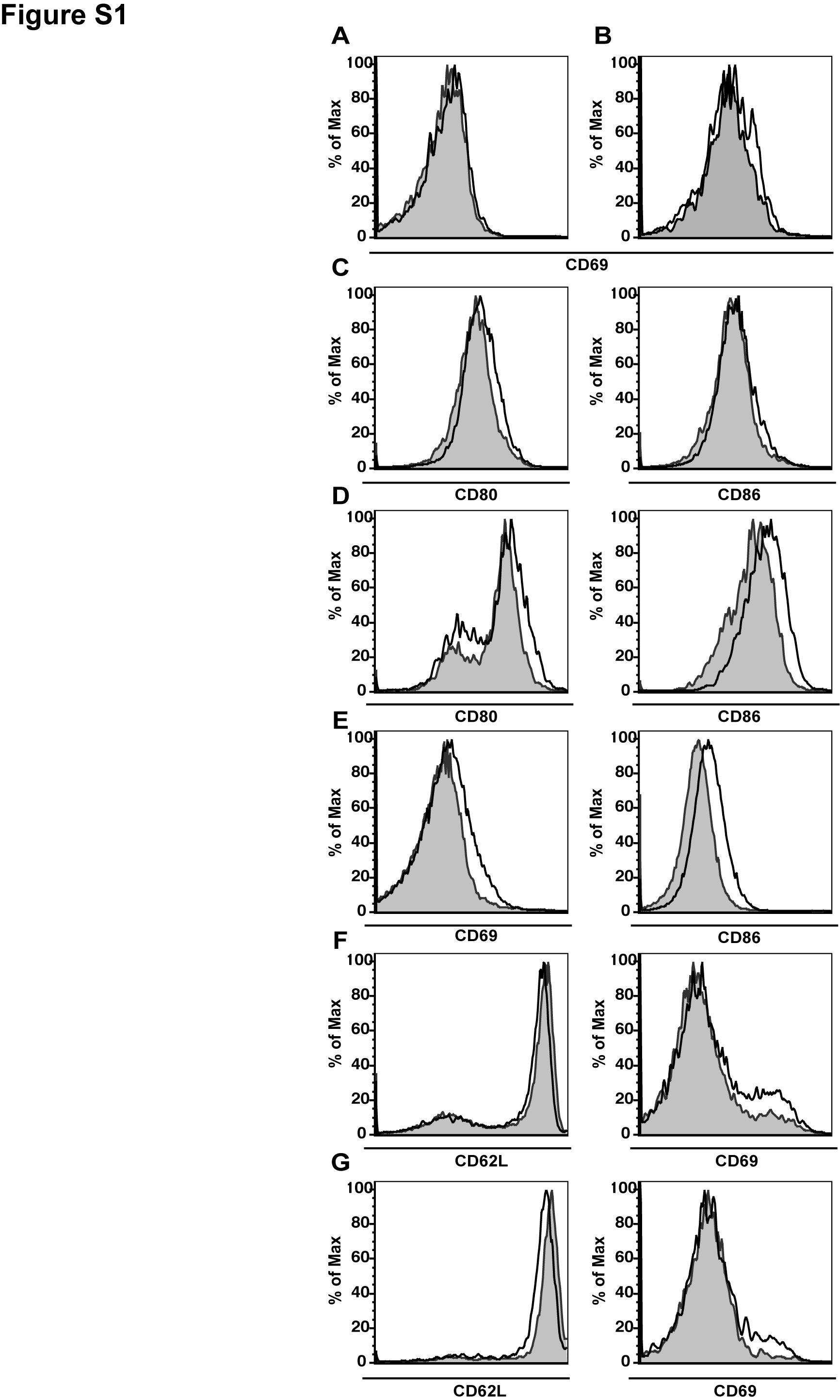

Supplement: Figure S1 — MyD88 deficiency decreases the capacity of cellular effectors to activate in response to CB4 infection. Representative histograms of expression of activation or costimulatory marker (as indicated) on the surface of A) NK cells (Pan-NK+, TCRβ −), B) NK T cells (Pan-NK+, TCRβ +), C) macrophages (CD11b+CD11c−), D) dendritic cells (CD11c+), E) B cells (CD19+), F) CD4+ T cells and G) CD8+ T cells from MyD88KO mice at day 4 post-infection with 400 pfu of CB4 (solid black lines) or mock-infection with DMEM (shaded histogram). Data is representative of at least 2 separate experiments. (0.70 MB TIF) [file pone.0004127.s001.tif]

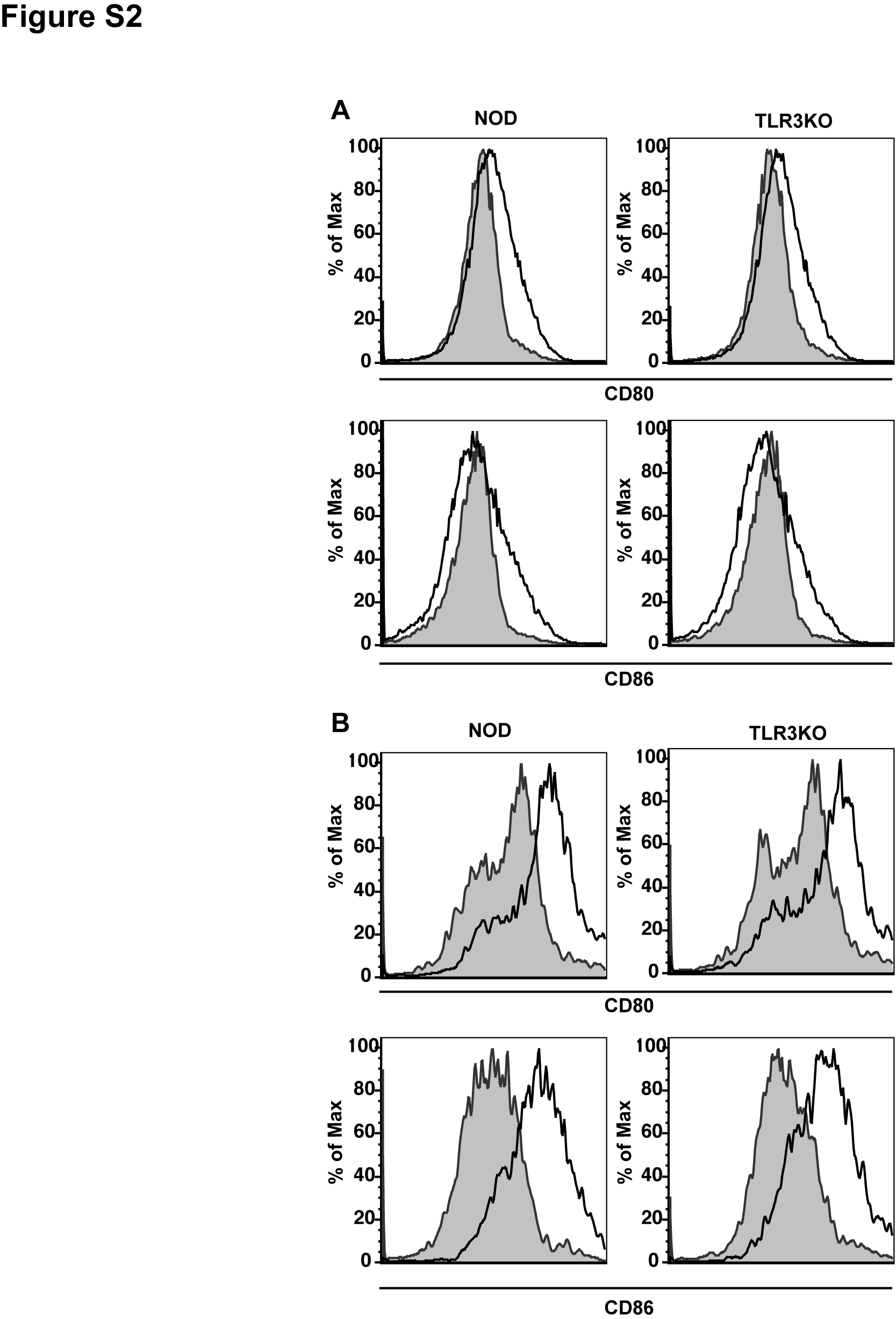

Supplement: Figure S2 — TLR3 deficiency does not affect the capacity of APCs to mature following LCMV infection. Representative histograms of CD80 and CD86 expression on the surface of (A) macrophages (CD11b+CD11c−) and (B) dendritic cells (CD11c+) from WT NOD (left panels) and TLR3KO (right panels) mice at 4 days post-infection with 1×10^5^ pfu of LCMV (solid black lines) or mock-infection with DMEM (shaded histogram). Data is representative of at least 2 separate experiments. (0.59 MB TIF) [file pone.0004127.s002.tif]

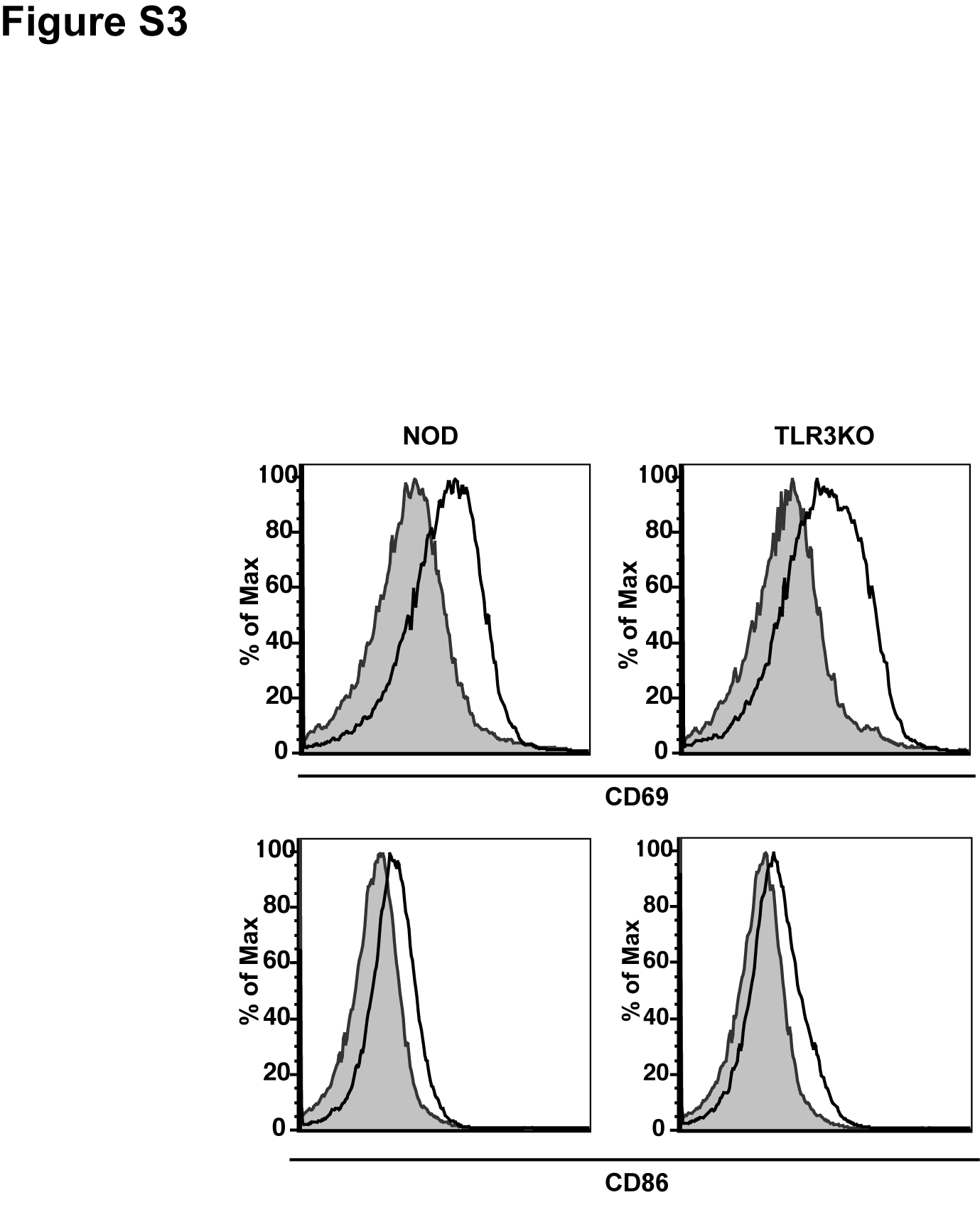

Supplement: Figure S3 — TLR3 deficiency does not affect the capacity of B cells to activate following CB4 infection. Representative histograms of CD69 and CD86 expression on the surface of B cells (CD19+) from WT NOD (left panels) and TLR3KO (right panels) mice at 4 days post-infection with 400 pfu of CB4 (solid black lines) or mock-infection with DMEM (shaded histogram). Data is representative of at least 2 separate experiments. (0.33 MB TIF) [file pone.0004127.s003.tif]

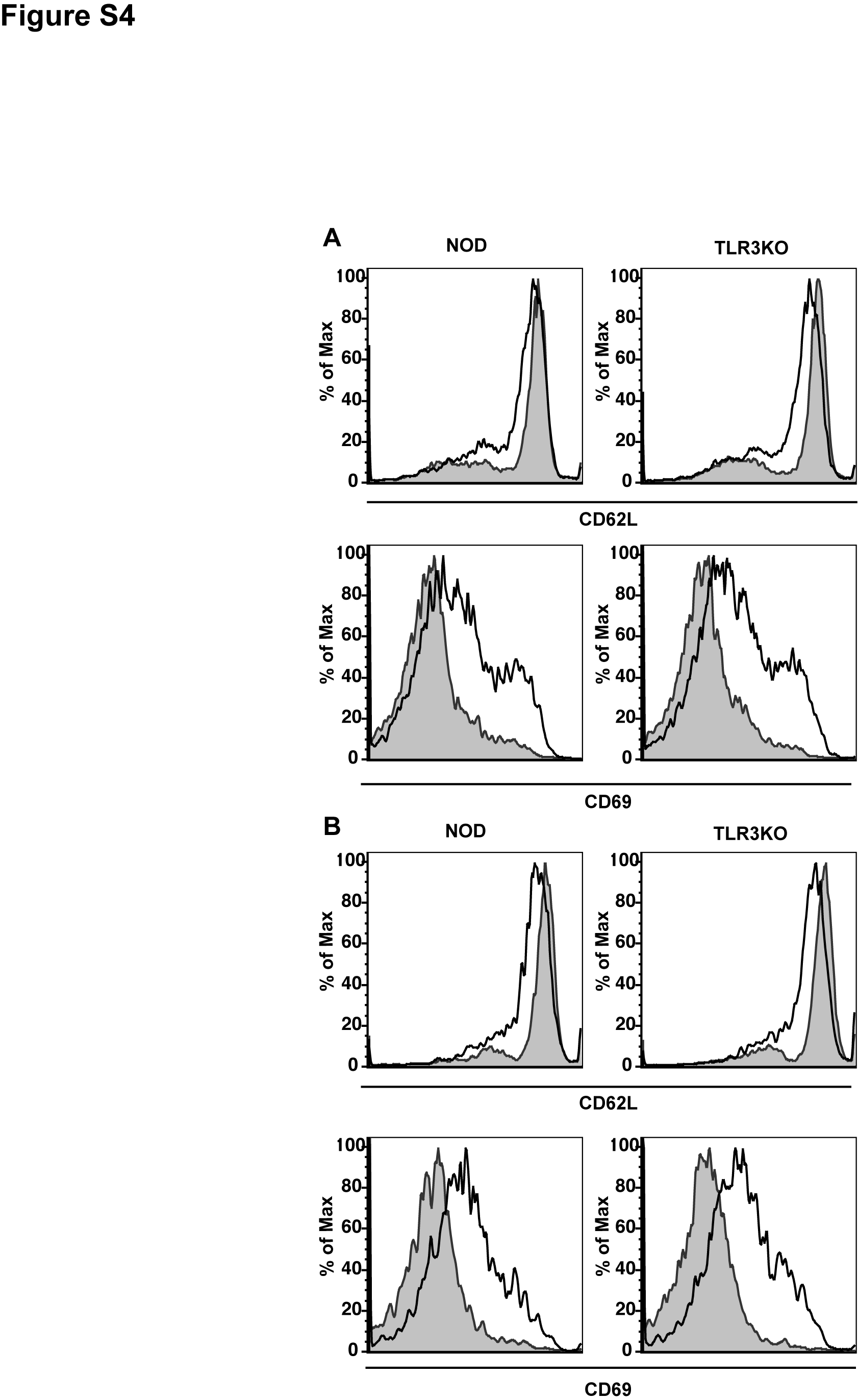

Supplement: Figure S4 — TLR3 deficiency does not affect the capacity of T cells to activate following LCMV infection. Representative histograms of CD62L and CD69 expression on the surface of (A) CD4 and (B) CD8 T cells from WT NOD (left panels) and TLR3KO (right panels) mice at 4 days post-infection with 1×10^5^ pfu of LCMV (solid black lines) or mock-infection with DMEM (shaded histogram). Data is representative of at least 2 separate experiments. (0.61 MB TIF) [file pone.0004127.s004.tif]

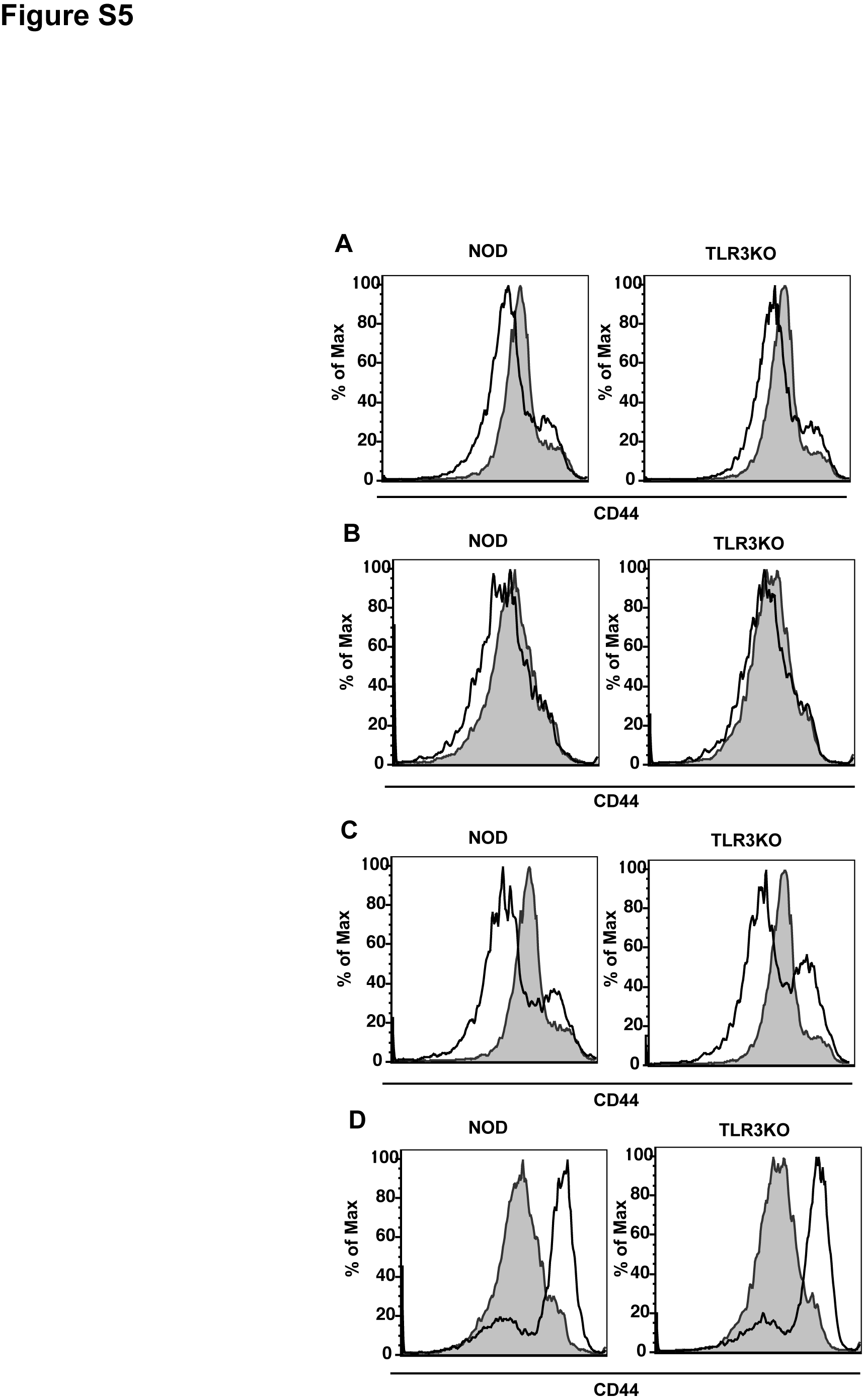

Supplement: Figure S5 — TLR3 deficiency does not affect T cell activation following LCMV or CB4 infection. Representative histograms of CD44 expression on the surface of (A, C) CD4 and (B, D) CD8 T cells from WT NOD (left panels) and TLR3KO (right panels) mice at 7 days post-infection with (A,B) 400 pfu of CB4 (solid black lines) or (C,D) 1×10^5^ pfu of LCMV (solid black lines) or mock-infection with DMEM (shaded histogram). Data is representative of at least 2 separate experiments. (0.61 MB TIF) [file pone.0004127.s005.tif]

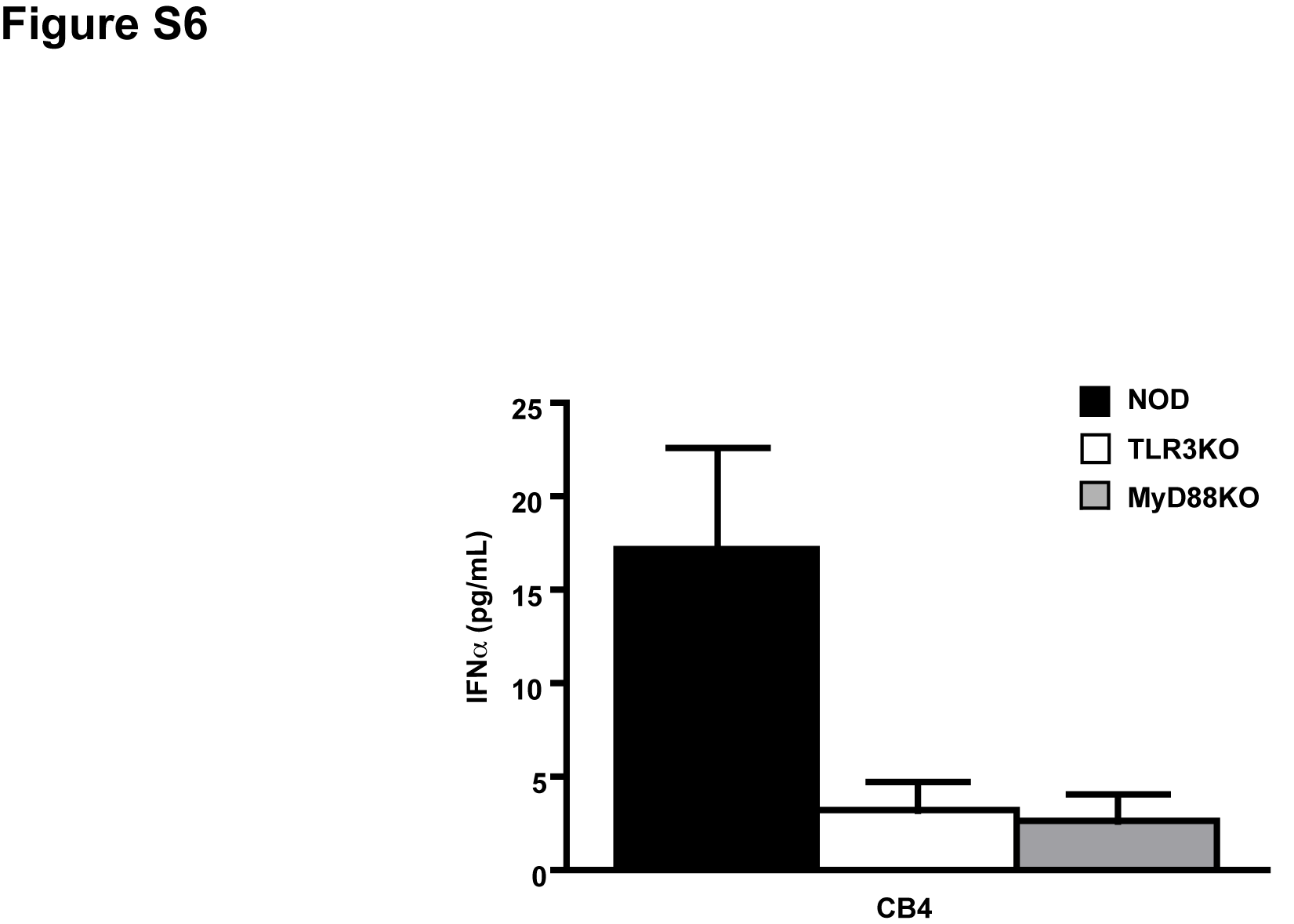

Supplement: Figure S6 — Type 1 interferon production is reduced following CB4 infection in both TLR3KO and MyD88KO mice. Serum levels of IFNα from WT NOD (black bars), TLR3KO (white bars) and MyD88KO (grey bars) mice were measured with a VeriKine Elisa Kit at 48 hours following infection with 400 pfu of N = at least 7 for each group. Pooled data from at least 2 independent experiments are presented as mean+/−s.e.m. (0.20 MB TIF) [file pone.0004127.s006.tif]
